# Supplementary material for: Comparing Depression Prevalence and Associated Symptoms with Intolerance of Uncertainty among Chinese Urban and Rural Adolescents: A Network Analysis
Source: Behav Sci (Basel). 2023 Aug 8;13(8):662. doi: 10.3390/bs13080662 (PMC10451834; doi:10.3390/bs13080662)
Supplement: Supplementary file 1 [file behavsci-13-00662-s001.zip › behavsci-2530054-supplementary.pdf]

Table S1. The edge weights matrix among all adolescence.

	PHQ1	PHQ2	PHQ3	PHQ4	PHQ5	PHQ6	PHQ7	PHQ8	PHQ9
PHQ1	0	0.353	0.056	0.295	0.045	0	0.141	0.067	0.014
PHQ2	0.353	0	0.155	0.120	0.067	0.180	0	0.080	0.077
PHQ3	0.056	0.155	0	0.281	0.114	0.036	0	0.014	0.087
PHQ4	0.295	0.120	0.281	0	0.156	0.036	0.188	0.070	-0.066
PHQ5	0.045	0.067	0.114	0.156	0	0.168	0.071	0.094	0.110
PHQ6	0	0.180	0.036	0.036	0.168	0	0.166	0.209	0.175
PHQ7	0.141	0	0	0.188	0.071	0.166	0	0.250	0.001
PHQ8	0.067	0.080	0.014	0.070	0.094	0.209	0.250	0	0.323
PHQ9	0.014	0.077	0.087	-0.066	0.110	0.175	0.001	0.323	0

Table S2. The edge weights matrix among adolescence in the urban.

	PHQ1	PHQ2	PHQ3	PHQ4	PHQ5	PHQ6	PHQ7	PHQ8	PHQ9
PHQ1	0	0.344	0.005	0.325	0.133	0	0.107	0	0.031
PHQ2	0.344	0	0.224	0.152	0.035	0.138	0	0.059	0.053
PHQ3	0.005	0.224	0	0.323	0.069	0.023	0	0.098	0.037
PHQ4	0.325	0.152	0.323	0	0.123	0.008	0.123	0.013	0
PHQ5	0.133	0.035	0.069	0.123	0	0.218	0.063	0.139	0.106
PHQ6	0	0.138	0.023	0.008	0.218	0	0.126	0.270	0.160
PHQ7	0.107	0	0	0.123	0.063	0.126	0	0.307	0.003
PHQ8	0	0.059	0.098	0.013	0.139	0.270	0.307	0	0.265
PHQ9	0.031	0.053	0.037	0	0.106	0.160	0.003	0.265	0

Table S3. The edge weights matrix among adolescence in rural.

	PHQ1	PHQ2	PHQ3	PHQ4	PHQ5	PHQ6	PHQ7	PHQ8	PHQ9
PHQ1	0	0.348	0.081	0.271	0.004	0.019	0.158	0.103	0
PHQ2	0.348	0	0.125	0.100	0.090	0.199	0	0.092	0.085
PHQ3	0.081	0.125	0	0.256	0.133	0.037	0	0	0.095
PHQ4	0.271	0.100	0.256	0	0.172	0.046	0.224	0.088	-0.071
PHQ5	0.004	0.090	0.133	0.172	0	0.141	0.076	0.077	0.113
PHQ6	0.019	0.199	0.037	0.046	0.141	0	0.174	0.172	0.180
PHQ7	0.158	0	0	0.224	0.076	0.174	0	0.215	0
PHQ8	0.103	0.092	0	0.088	0.077	0.172	0.215	0	0.339
PHQ9	0	0.085	0.095	-0.071	0.113	0.180	0	0.339	0

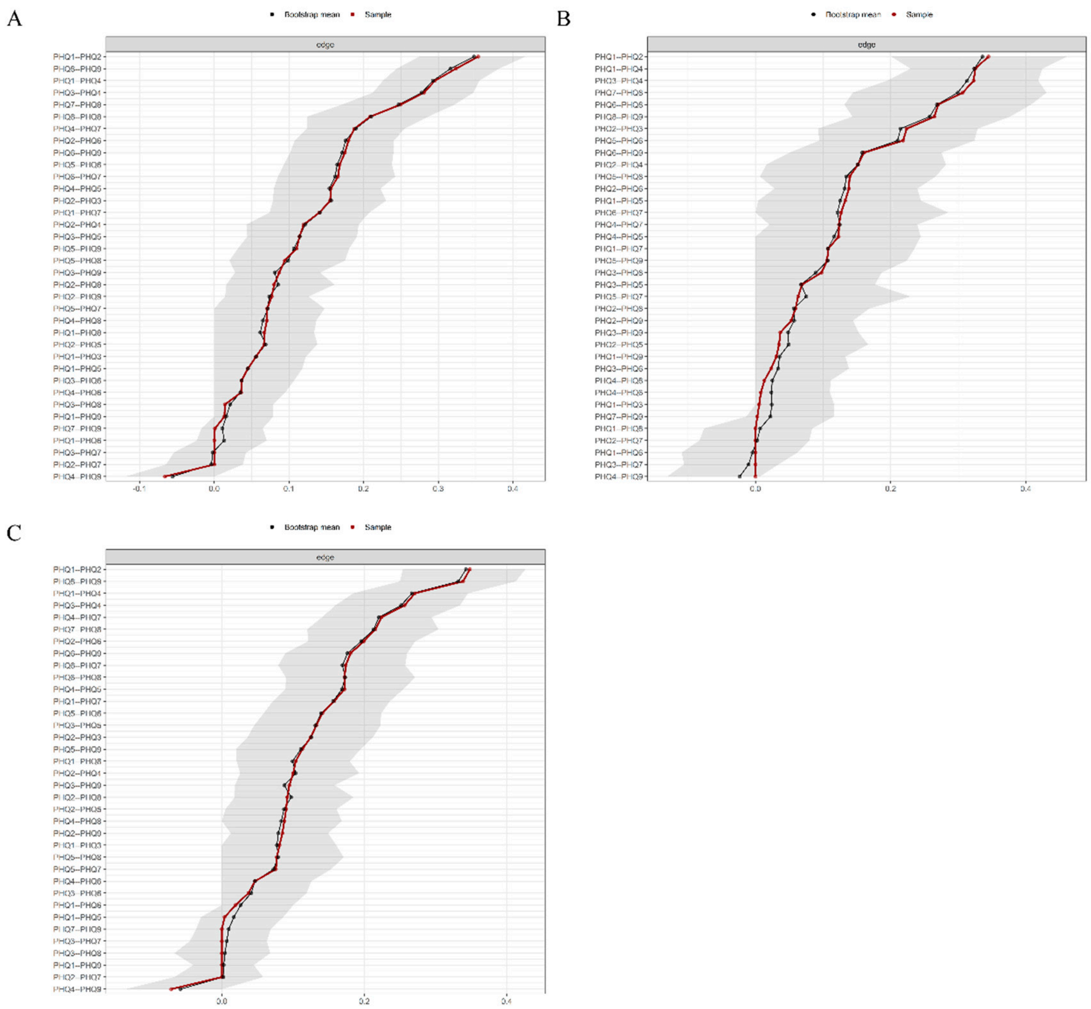

Figure S1. Nonparametric bootstrapped confidence intervals of estimated edges. The red line represents the estimated edge, while the dark area indicates the 95% bootstrap confidence interval. A indicates all adolescents. B indicates adolescence in the urban. C indicates adolescence in rural.

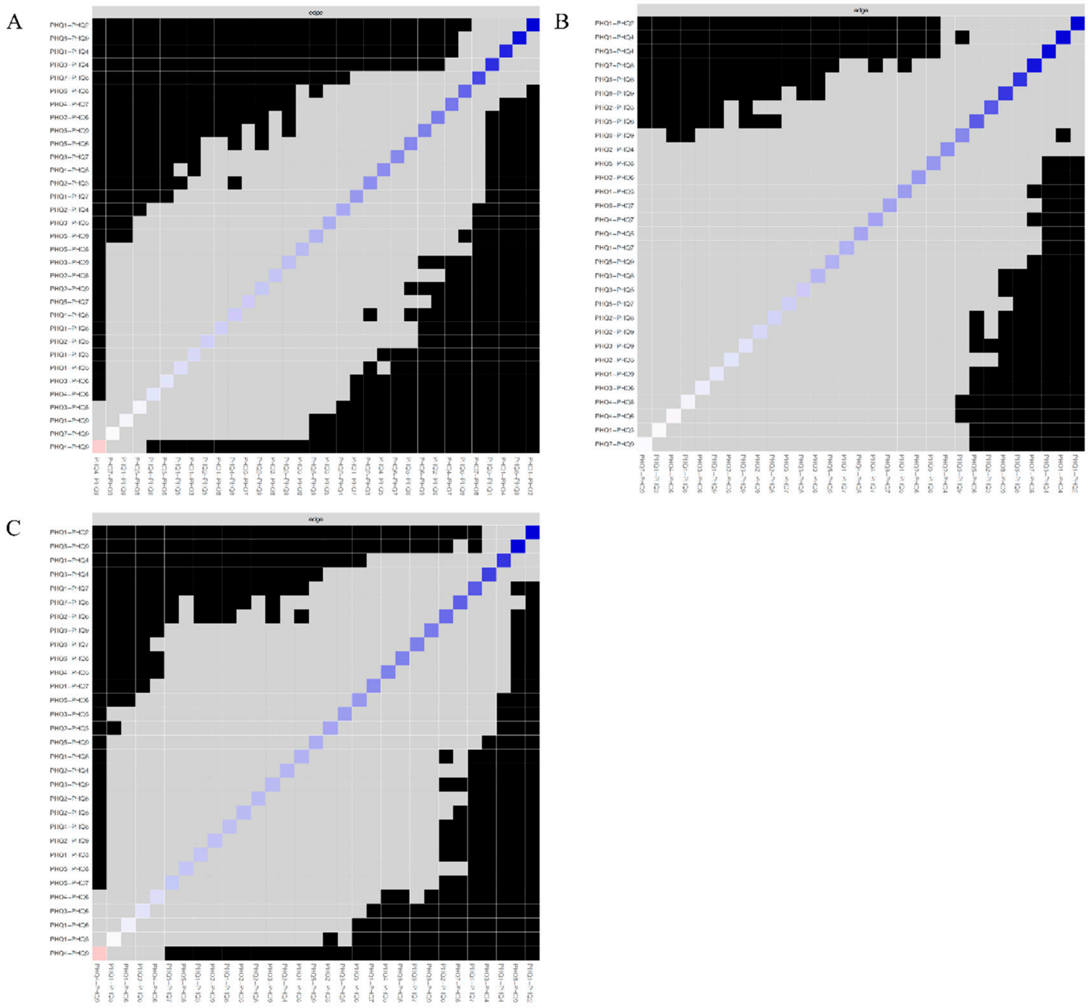

Figure S2. Bootstrapped stability test for edge-weight. The results of the bootstrapped difference tests (α=0.05) for edge-weights were shown in this figure. The color of the boxes indicates whether edge-weights differ significantly from each other (i.e., black) or do not differ significantly (i.e., grey). The diagonal line indicates the strength of edge-weights, shifting from red (negative associations) to white (representing weaker edges) and ultimately blue (representing stronger edge-weights). A indicates all adolescents. B indicates adolescence in the urban. C indicates adolescence in rural.

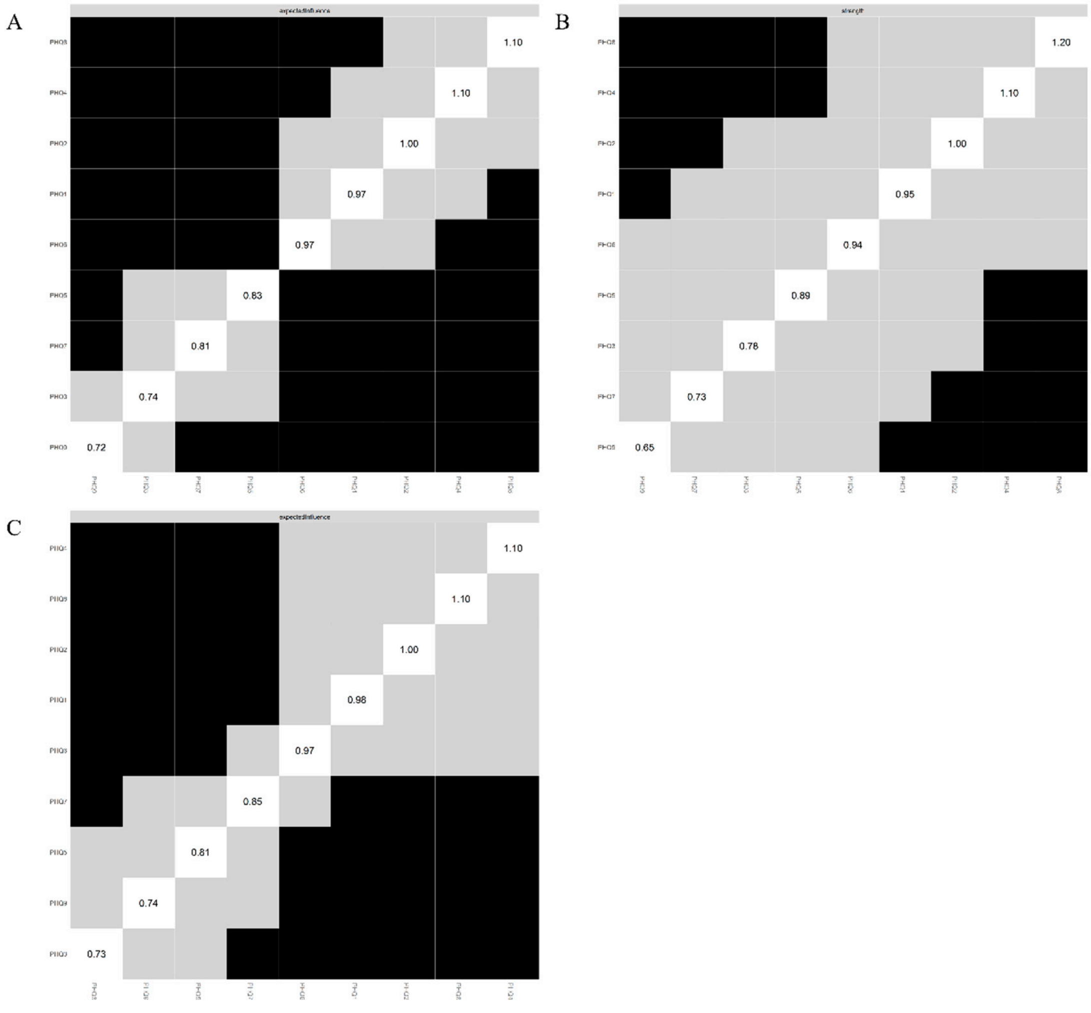

Figure S3. Bootstrapped stability test for edge-weight. The results of the bootstrapped difference tests (α=0.05) for edge-weights were shown in this figure. The colour of the boxes indicates whether edge-weights differ significantly from each other (i.e., black) or do not differ significantly (i.e., grey). A indicates all adolescents. B indicates adolescence in the urban. C indicates adolescence in rural.
